# Associations Between 24 H Movement Behaviors and Body Weight in Postpartum Women: An Isotemporal Substitution Model Approach

**DOI:** 10.3390/clockssleep8010012

**Published:** 2026-03-07

**Authors:** Erin E. Kishman, Shawn D. Youngstedt, Xuewen Wang

**Affiliations:** 1 Department of Exercise Science, School of Public Health, University of South Carolina, Columbia, SC 29208, USA; 2 BioFrontiers Center, University of Colorado Colorado Springs, Colorado Springs, CO 80918, USA; 3 Edson College of Nursing and Health Innovation, Arizona State University, Phoenix, AZ 85004, USA

**Keywords:** twenty-four-hour movement behaviors, sleep, physical activity, postpartum

## Abstract

Background/Objectives: There are limited data on the dynamic changes in daily composition of movement behaviors (sleep; moderate-to-vigorous physical activity, MVPA; light physical activity, LPA; and sedentary time, SED) and their associations with body weight in postpartum women. The purpose of this study was to examine associations of reallocating time in one behavior to another with body weight, at different times in the first year postpartum. Methods: The study included 86 women who delivered a singleton infant at ≥37 weeks gestation. Physical activity and sleep were measured via actigraphy in early, mid-, and late postpartum. Body weight was measured at each timepoint. Isotemporal substitution models were used to examine the association of reallocating ten minutes of one behavior (MVPA, LPA, SED, or sleep) to another, with body weight. Results: Participants spent most of their day in SED (~52–53%), followed by sleep (~30%), LPA (~12–13%), and then MVPA (~2%) throughout the first year postpartum. In early and mid-postpartum, but not late postpartum, reallocating 10 min of MVPA to LPA, SED, or sleep was associated with lower body weight (range: 3.07–4.03 kg lower). In early and late postpartum, reallocating 10 min of SED to LPA was associated with a lower body weight (4.03 kg and 1.04 kg, respectively). In participants who slept ≥7 h per day, reallocating sleep to LPA in early postpartum, and MVPA time to LPA in mid-postpartum was associated with lower body weight. In those who slept <7 h, no significant associations with body weight were found when reallocating time from one behavior to another. Conclusions: Encouraging LPA throughout the postpartum period may be beneficial for weight loss, and having enough sleep may be especially important for early to mid-postpartum. Future research examining the impact of changes in LPA on body weight in the postpartum period are needed, along with postpartum specific 24 h movement guidelines.

## 1. Introduction

In a 24 h day, individuals can partake in four main types of movement behaviors: sedentary behavior (SED), light physical activity (LPA), moderate-to-vigorous physical activity (MVPA), and sleeping. These four behaviors have individually been associated with changes in body weight and obesity [1,2,3]. However, increasing time spent in one behavior will displace time spent in another behavior, making it important to examine all four behaviors together in the 24 h activity cycle (24-HAC) [4]. Countries, such as Canada, have implemented 24 h movement guidelines [5]. For adults, they recommend less than 8 h of sedentary behavior a day, ≥150 min of moderate-to-vigorous physical activity a week, sleeping 7–9 h a night, and several hours of light physical activity. Meeting the guidelines is associated with a lower risk of weight gain and cardiometabolic diseases.

In the postpartum period, women experience changes and new barriers to physical activity, such as caring for an infant, that may affect time spent in each of the 24-HAC behaviors [6]. There is evidence of changes in time spent in SED, LPA, MVPA, and sleep during the postpartum period, but there have been limited studies that examine all four behaviors during the postpartum period [7,8,9,10,11,12]. Since the behaviors of the 24-HAC are related to health outcomes, examining the 24-HAC in postpartum women will give insight into how women are spending their day throughout the postpartum period and if there are potential areas to intervene in order to improve health outcomes.

Additionally, many women are not returning to their pre-pregnancy weights within the first year postpartum, which may put them at risk for obesity in the future [13,14,15,16]. Obesity is associated with increased health risks including cardiometabolic diseases, adverse pregnancy outcomes, and psychological consequences such as depression and low self-esteem [17,18]. Physical activity and sleep duration have been associated with weight changes in the postpartum period. One study found that higher levels of LPA and total physical activity levels at 3 months postpartum were associated with lower postpartum weight retention at 6 and 12 months postpartum [19]. Sleep restriction is associated with changes in hunger and appetite and may increase the risk of obesity and metabolic diseases [20]. Short sleep duration during the postpartum period has been associated with greater adiposity and weight retention [21,22]. In adult and adolescent populations, the composition of the 24-HAC has been associated with body weight outcomes [23,24]. However, no study has examined the associations of reallocating time from one behavior in the 24-HAC to another behavior with changes in body weight in postpartum women. Since the postpartum period can be a time of body weight change, examining the associations of reallocating behaviors of the 24-HAC with body weight may provide insight into what behaviors provide the greatest benefit for reducing weight retention. The purpose of this study is to examine the associations of reallocating time spent in one of the 24-HAC behaviors to another 24-HAC behavior with body weight during first year postpartum.

## 2. Results

### 2.1. Participant Characteristics

Eighty-six participants had valid sleep and activity data at early, mid-, and late postpartum. At the mid timepoint, four participants had data from 4 months postpartum and 82 had data from 6 months postpartum. At the late timepoint, 17 participants had data from 9 months postpartum, and 69 participants had data from 12 months postpartum. Participant characteristics are displayed in Table 1.

### 2.2. Time Allocation in Each Behavior

The percentage of time spent in sedentary time, LPA, MVPA, and sleep is displayed in Table 2. At all three timepoints, SED accounted for 51–52% of 24 h, followed by sleep which accounted for approximately 30%. LPA accounted for 12–13% and MVPA accounted for the smallest proportion of approximately 2%. The number of participants meeting the Canadian 24 h movement guidelines at each timepoint are displayed in Table 3. At the early and mid-timepoints, no participants met all Canadian 24 h movement guidelines. During late postpartum, one participant met all three Canadian 24 h movement guidelines.

### 2.3. Cross-Sectional Isotemporal Substitution Models (ISM) by Timepoint

**Figure 1:**
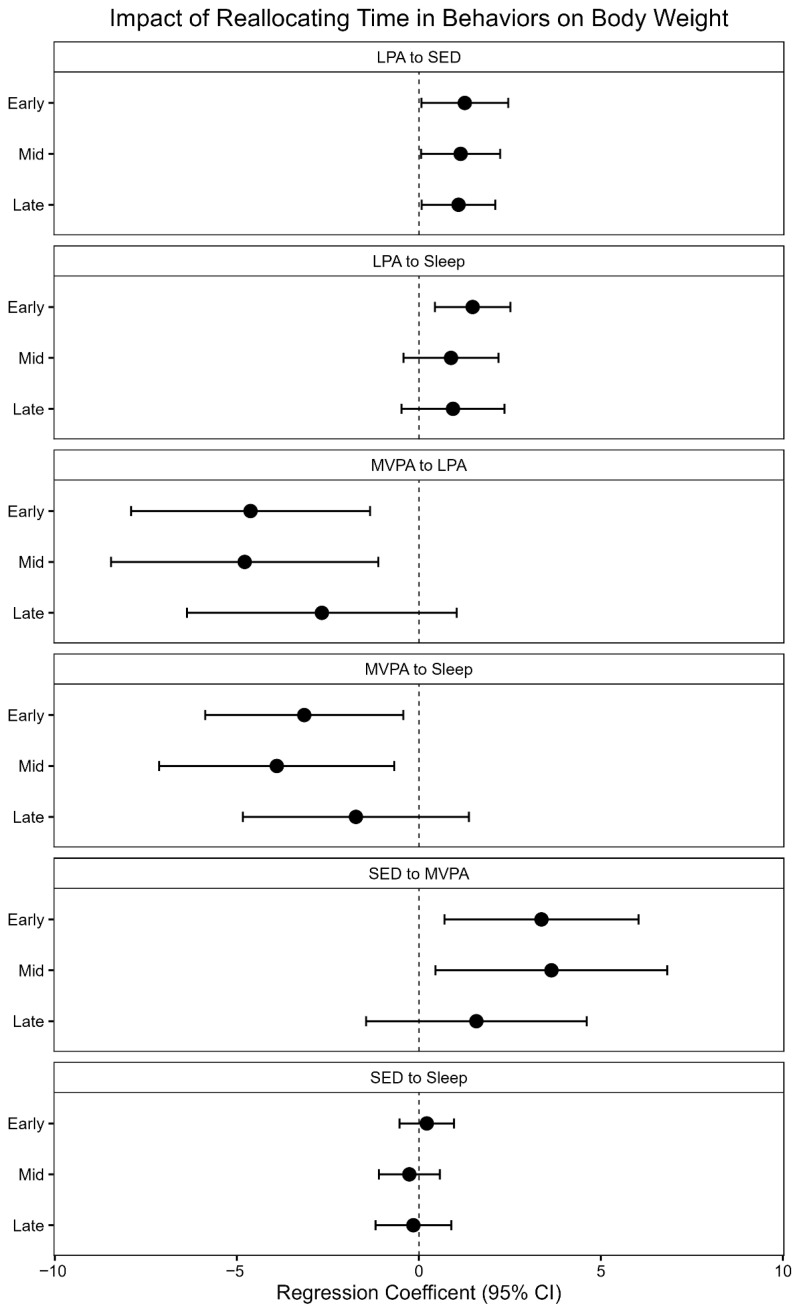
Impact of reallocating 10 min of one behavior to another on body weight (kg) at early, mid-, and late postpartum. The regression coefficients are from Isotemporal Substitution Models at each time point and represent the estimated change in body weight (kg) for a 10 min substitution of one behavior for another behavior.

Results from the cross-sectional ISM are displayed in Figure 1. The reallocations are symmetrical (for example: reallocating time from sleep to LPA or LPA to sleep will result in the same estimate, but in the opposite direction), therefore only one direction of the reallocation is displayed in the figure. Results are reported as body weight change (95% Confidence Interval (CI)) with reallocating 10 min from one behavior to another. Body weight change is the regression coefficient from the Isotemporal Substitution Model.

#### 2.3.1. Early Postpartum

In early postpartum, reallocating 10 min of MVPA, SED, or sleep to LPA was associated with a 4.6 kg, 1.3 kg, and 1.5 kg lower body weight (95% CI: −7.9, −1.3, *p* = 0.0064; CI: −2.5, −0.07, *p* = 0.0387; CI: −2.5, −0.4, *p* = 0.0058, respectively). Reallocating 10 min of LPA, SED, or sleep to MVPA was associated with a 4.6 kg, 3.4 kg, and 3.2 kg higher body weight (CI: 1.3, 7.9, *p* = 0.0064; CI: 0.7, 6.0, *p* = 0.014; CI: 0.4, 5.9, *p* = 0.0238). Reallocating 10 min of LPA or MVPA to SED was associated with a 1.3 kg higher or 3.4 kg lower body weight (CI: 0.06, 2.5, *p* = 0.0387; CI: −6.0, −0.7, *p* = 0.014). Lastly, reallocating LPA or MVPA to sleep was associated with a 1.4 kg higher and 3.2 kg lower body weight (CI: 0.4, 2.5, *p* = 0.0058; CI: −5.9, −0.4, *p* = 0.0238), respectively. The R^2^ for the early postpartum models was 0.22 (*p* = 0.011).

#### 2.3.2. Mid-Postpartum

In mid-postpartum, reallocating 10 min of MVPA or SED to LPA was associated with a 4.8 kg and 1.1 kg lower body weight (CI: −8.5, −1.1, *p* = 0.0185; CI: −2.2, −0.06, *p* = 0.039). Reallocating 10 min of LPA, SED, or sleep to MVPA was associated with a 4.8 kg, 3.6 kg, and 3.9 kg higher body weight (CI: 1.1, 8.5, *p* = 0.0113; CI: 0.5, 6.8, *p* = 0.0256; CI: 0.7, 7.1, *p* = 0.0185). Reallocating 10 min of LPA or MVPA to SED was associated with a 1.1 kg higher and 3.6 kg lower body weight (CI: 0.06, 2.2, *p* = 0.039; −6.8, −0.5, *p* = 0.0256). Lastly, reallocating 10 min of MVPA to sleep was associated with a 3.9 kg lower body weight (CI: −7.1, −0.7, *p* = 0.0185). The R^2^ for the mid-postpartum models was 0.21 (*p* = 0.013).

#### 2.3.3. Late Postpartum

In late postpartum, reallocating 10 min of SED to LPA was associated with a 1.1 kg lower body weight (CI: −2.1, −0.08, *p* = 0.0353), and conversely, reallocating 10 min of LPA to SED was associated with a 1.1 kg higher body weight (CI: 0.08, 2.1, *p* = 0.0353). The R^2^ for the late postpartum models was 0.21 (*p* = 0.017).

#### 2.3.4. Isotemporal Substitution Models (ISM) with Physical Activity

Due to limitations with using cut points to determine LPA and MVPA from actigraphy, we also examined the ISMs using a combined physical activity (PA) variable. However, when only PA, SED, and sleep were in the models, there were no significant associations when reallocating time from one behavior to another.

#### 2.3.5. Isotemporal Substitution Models (ISM) for Short and Normal Sleep Duration

It is recommended that adults sleep 7–9 h per day for optimal health [25]. Therefore, we ran additional ISMs in participants who slept <7 h per day and those who slept ≥7 h. The number of short sleepers (<7 h per day) was 35 in early, 38 in mid-, and 33 in late postpartum. In short sleepers, there were no significant associations when reallocating time from one behavior to another.

**Figure 2:**
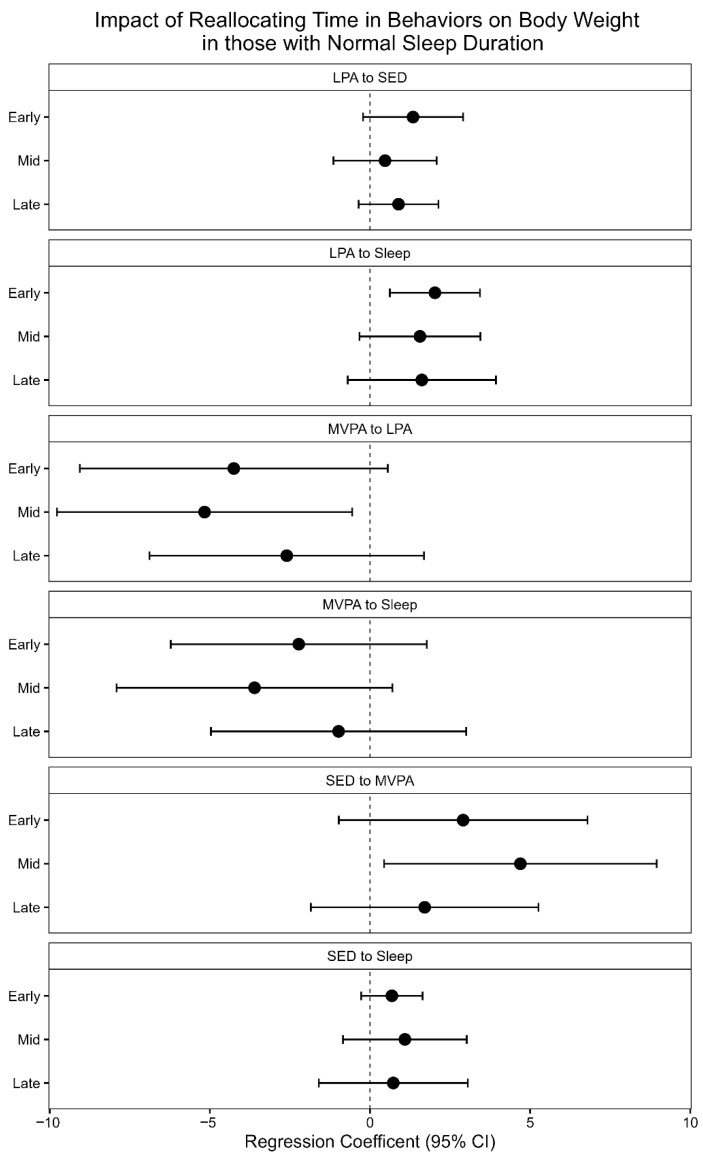
Impact of reallocating 10 min of one behavior to another on body weight (kg) at early, mid-, and late postpartum in those sleeping ≥7 h a day. The regression coefficients are from Isotemporal Substitution Models at each time point and represent the estimated change in body weight (kg) for a 10 min substitution of one behavior for another behavior.

For participants with normal sleep durations (≥7 h per day), results are displayed in Figure 2. In early postpartum, reallocating 10 min of sleep to LPA was associated with a 2.0 kg lower body weight (CI: −3.4, −0.6, *p* = 0.0058). In mid-postpartum, reallocating 10 min of LPA or SED to MVPA was associated with a 5.2 kg and 4.7 kg higher body weight (CI: 0.6, 9.8, *p* = 0.029; CI: 0.4, 9.0, *p* = 0.0315), respectively. There were no significant associations during late postpartum. R^2^ for early, mid, and late models were 0.25, 0.33, and 0.17, respectively (*p* = 0.114, 0.030, and *p* = 0.365, respectively).

## 3. Discussion

This study provides objectively measured descriptive data on the behaviors comprising the 24-HAC over the first year postpartum and the associations of reallocating behaviors with body weight. To our knowledge, this is the first study to examine the associations of reallocating 24-HAC behaviors with body weight at different timepoints in the postpartum period.

Throughout the postpartum period, participants spent over half their day in SED, ~30% of their day sleeping, ~12–13% in LPA, and ~2% in MVPA. Recently, Canadian guidelines for physical activity, sedentary behavior, and sleep throughout the first year postpartum were published [26]. These guidelines provide recommendations for engaging in physical activity by gradually increasing physical activity levels based on healing and contraindications and recommend at least 120 min of MVPA a week. However, there have yet to be optimal sleep time and LPA recommendations for the postpartum period, and the United States do not currently have 24 h movement guidelines; so, we will compare the results of our participants to the Canadian 24 h movement guidelines.

Considering the Canadian 24 h movement guidelines, a little over half the participants met the guideline for sleeping 7–9 h a night at each timepoint; the majority of participants met the guidelines for 150 min of MVPA per week, but no participants met the guidelines for <8 h of sedentary behavior in early and mid-postpartum [5]. Only two participants met the sedentary time guideline during late postpartum. This highlights that decreasing sedentary behavior may be an area to target for future interventions. Additionally, only one participant met all three guidelines during late postpartum. While this emphasizes room for improvement in meeting the guidelines, it is also important to point out that these guidelines are for the general adult population. More research is needed to determine optimal sleep duration and the impact of LPA during the postpartum period to develop postpartum-specific 24 h movement behavior guidelines [26].

The findings from this analysis suggest increasing time in LPA may be beneficial for weight maintenance or loss during the postpartum period. Reallocating time from MVPA to LPA during early and mid-postpartum and reallocating time from SED to LPA during early, mid-, and late postpartum were all associated with lower body weight. Another study found similar results in overweight and obese adults where reallocating SED to LPA was associated with a lower waist circumference [27]. Furthermore, a study in postpartum women previously found that higher levels of total PA and LPA in early postpartum was associated with less postpartum weight retention at one year postpartum [19].

Nevertheless, our results for MVPA are intriguing. The average MVPA in our study was approximately 175, 191, and 232 min per week at early, mid-, and late postpartum. This is above the recommended minimum of 150 min MVPA per week for the general population. Reallocating time from LPA, SED, and sleep to MVPA were all associated with a higher body weight in early and mid-postpartum, but not late postpartum. A systematic review of studies in adult populations found reallocating time to MVPA to be associated with lower body mass index, waist circumference, and body fat percentage [28]. However, the direction of the association of MVPA with body weight in our results is unclear. There may be behavioral changes leading to changes in physical activity and body weight that were unmeasured. For example, an individual who has lost less weight during postpartum may be more likely to participate in MVPA. Additionally, there is evidence that total daily energy expenditure does not increase equally with an increase in physical activity energy expenditure [29,30]. Some individuals may participate in more sedentary behavior after participating in exercise, leading to a lower non-physical activity energy expenditure, and potentially impacting body weight. Nevertheless, MVPA is likely beneficial for other health outcomes, and as the Canadian postpartum guidelines recommend, women who return to exercise within 12 weeks after delivering are likely to experience psychological benefits, improved sleep quality, and cardiometabolic benefits [26]. Our results suggest that the influence of MVPA on weight may be changing over time during the postpartum period, and that additional MVPA in this population who had already spent an average of 175–232 min per week on MVPA is unlikely to lead to greater weight loss.

Interestingly, different results were found for individuals whose sleep was <7 h versus those ≥7 h a night. In those sleeping <7 h a day, no significant association with weight was found for any reallocation of time. In those sleeping ≥7 h a night, reallocating LPA or SED to MVPA in mid-postpartum was associated with higher body weights, and reallocating sleep to LPA in early postpartum was associated with lower body weight. This may indicate that achieving the recommended 7–9 h of sleep a day is needed for the reallocation of the other behaviors to impact body weight. There is also evidence that the time-of-day MVPA is performed can impact sleep outcomes [31]. Examining when MVPA is performed in relation to sleeping and if sleep time is being shortened in order to participate in MVPA is needed to better understand the relationship between sleep and MVPA in the postpartum period. More research in a larger sample of postpartum women is necessary to examine if this finding is transient and specific to the postpartum period.

Additionally, there are not validated cut points for physical activity in postpartum women. We used the Troiano et al. [32] cut points, which have been validated in adults and used previously in postpartum women. However, studies that have compared different physical activity cut points in postpartum women have found large differences in the amount of MVPA and LPA depending on what cut points were used [8,11,33]. Using different physical activity cut points may impact the results of this study. However, our results indicate that the time allocated to LPA and MVPA does matter.

Currently, the physical activity guidelines for the postpartum period are not specific to timing after delivery. These results demonstrate a need to further investigate the behaviors of the 24-HAC, specifically during the postpartum period, to develop guidelines for optimal health. It may be difficult for women to achieve the recommended sleep guidelines during postpartum since short sleep may be due to the infant’s sleep pattern, but providing specific SED, LPA, and MVPA guidelines may be beneficial to improve health outcomes in mothers. The results from the ISM models suggest engaging in LPA and reducing SED may be beneficial for weight loss during early, mid-, and late postpartum. Additionally, since many participants were spending greater than 50% of their day sedentary, replacing SED with LPA may be a potential area to intervene.

The strengths of this study include the objective assessment of the 24-HAC in a cohort of women from 6 to 8 weeks to 12 months postpartum. Previous ISM literature has not included objectively measured sleep in their analyses [28], making the inclusion of objectively determined sleep a major strength of the study. While the inclusion of actigraphy is a strength of this study, actigraphy does have limitations. For sleep measures, actigraphy has low specificity in detecting wakefulness during the sleep period and may overestimate total sleep time [34,35]. There is also evidence actigraphy is better at classifying MVPA than LPA [36]. Furthermore, while participants were instructed to wear the Actiwatch all day and night and the Actigraph during waking hours, some participants had non-wear time and therefore their total time in all four behaviors did not equal 24 h. It is unknown what behaviors participants were partaking in when the Actigraphs were removed. As discussed earlier, there are not validated cut points for physical activity in postpartum women and the use of different cut points could impact the results of this study.

Additionally, results of our study should be interpreted with caution as this was in a small sample of postpartum women and an exploratory analysis. Due to the small sample size, we were not able to adjust for or examine all relevant factors related to body weight in the postpartum period such as breastfeeding, socioeconomic status, gestational weight gain, and pre-pregnancy weight and fitness levels. Replication of these findings in a larger sample is needed as our results could be influenced by the small sample size. Future studies are needed to determine the feasibility of reallocating time from one behavior to another during the postpartum period. Additionally, our sample may not be generalizable to other postpartum populations. Lastly, ISM findings are not a true temporal substitution and may represent more population-level changes in behavior [37].

## 4. Materials and Methods

### 4.1. Participants and Study Design

Data are from a prospective cohort study that examined trajectories of body weight, body fat, and sleep parameters in Black and White women from 6 to 8 weeks to 12 months postpartum [12,38]. Women were from the Columbia metropolitan area in South Carolina and were recruited from October 2018 to January 2020. Measures took place during early (6–8 weeks), mid- (6 months), and late postpartum (12 months). If participants did not have 6- or 12-month data available, 4- or 9-month data was used if available, respectively. The inclusion criteria for the study included women ≥18 years of age, delivered a singleton infant at ≥37 weeks gestation, self-identified as Black or White, and were providing care for the infant [11]. Women were excluded if they self-reported diseases or medications that may affect body weight or sleep, such as thyroid disease, diabetes, sleep apnea, clinical depression, and the use of the contraceptive Depo-Provera. The study protocol was approved by the Institutional Review Board of the University of South Carolina, Columbia, SC (Pro00076434). All participants signed an informed consent form prior to participation.

### 4.2. Measurements

#### 4.2.1. Physical Activity and Sedentary Behavior

Activity was objectively measured using actigraphy (GT3X+; Actigraph, Pensacola, FL, USA). At each timepoint, participants were instructed to wear the GT3X+ on their hip for 7 days. Participants removed the monitor for sleep and water activities and were instructed to record when the monitor was removed and put back on. Data were collected at a sampling rate of 30 Hz in 10 s epochs and were analyzed in the manufacturer-provided software (ActiLife software version 6.13.4). Non-wear time was excluded using the Choi algorithm available in ActiLife, which defines 90 consecutive minutes of zero counts as non-wear [39]. A valid day of wear time was defined as 600 or more minutes of accumulative wear [32]. Sedentary behavior, LPA, and MVPA were determined using the Troiano et al. [32] cut points as follows: Sedentary 0–99, Light 100–2019, Moderate 2020–5998, and Vigorous > 5999 counts per minute. At least 3 valid days of wear time were required [40] to be included in this analysis.

#### 4.2.2. Total Sleep Time

A different Actigraphic monitor was used to measure total sleep time in a 24 h period (Actiwatch Spectrum Plus: Phillips Respironics; Bend, OR, USA). Participants were instructed to wear the monitor on their nondominant wrist for 7 days (same as activity monitor) at each timepoint and were instructed to only remove it for activities in saltwater. The Actiwatch has a small event marker button that participants were instructed to press when they were trying to fall asleep and when they woke up. Participants were also instructed to fill out a daily sleep log during the same 7 days. Participants recorded the time they got into bed, time trying to sleep, time waking up, time out of bed, and nap times. The manufacturer’s software (Philips Actiware 6) was used to analyze the actigraphy data in 30 s epochs. A standardized approach using a hierarchical ranking of inputs was used to determine time in bed [event markers, sleep diary, light intensity, and activity counts] [41,42]. Rest intervals for naps were created if a diary or event marker indicated an attempt to sleep by the participant and minor rest intervals were auto-calculated by the software to catch potential naps not recorded by the event markers or sleep log. The minimum rest interval size was set to 15 min with high sensitivity. Once time in bed was determined, the software provided total sleep time. All sleep bouts that occurred in a 24 h period, including the nighttime sleep interval and naps, were included in total sleep time. Participants with at least 3 nights of wear were included in the analysis, as previous research has found 3 nights of sleep are needed when examining the means of characteristics [43].

#### 4.2.3. Body Weight

Body weight was measured at each timepoint using a calibrated digital scale (Health O Meter^®^ Professional, Pelstar LLC, McCook, IL, USA). Women wore standard scrubs and were without shoes. Weight was taken two times and measured to the nearest 0.1 kg. If the two measurements varied by more than 0.1 kg, a third measure was taken. The average of the two weights, if different, were used for analysis.

#### 4.2.4. Statistical Analysis

Descriptive statistics, such as means, standard deviations, and proportions were calculated. Daily means for each behavior (SED, LPA, MVPA, and sleep) were divided by 24 h to calculate the percentage of time spent in that behavior in a 24 h day at early, mid-, and late postpartum. To examine the associations of body weight with reallocating time from one behavior to another, isotemporal substitution models (ISM) were performed. The ISM includes a variable for total time, which equals the amount of time spent in sleep (total sleep time) + SED + LPA + MVPA, which is roughly 24 h for each individual [44]. An example of an ISM for when SED is omitted is below:

Body Weight = (β_1_) Sleep + (β_2_) LPA + (β_3_) MVPA + (β_4_) Total Time + ε

In this model with SED removed, the influence of SED is distributed to other behaviors. Thus, the coefficients for the other behaviors represent the effect of reallocating 1 min of that behavior in place of SED, with all other variables held constant. ISMs were performed for early, mid-, and late postpartum with sleep, SED, LPA, and MVPA as independent variables in the model. Additional models were run with MVPA and LPA combined into a total physical activity variable in the model. All models were adjusted for age, race, parity, and wake after sleep onset (in minutes). It is recommended to adjust for wake after sleep onset when examining the 24-HAC because wake after sleep onset is different from daytime sedentary or activity behavior; it is not a behavior one chooses to partake in, but is a component of the 24 h day [45]. ISM results are displayed in 10 min reallocations. Statistical significance was set to *p* < 0.05 and all analyses were performed in SAS 9.4 (SAS institute Inc., Cary, NC, USA). Figures were made in R 4.4.0 using ggplot2 [46,47].

## 5. Conclusions

In conclusion, when comparing to the Canadian 24 h Movement Guidelines, postpartum women in this sample had much higher SED than recommended. Throughout postpartum, reallocating time to LPA and reducing SED may be beneficial for weight maintenance or loss. However, the relative importance of the 24-HAC behaviors may change throughout the first year postpartum. Sleeping more than 7 h may be especially important in early to mid-postpartum. Future research examining the associations of the 24-HAC with health outcomes and development of guidelines more specific to duration after childbirth is needed.

## Figures and Tables

**Table 1:** Participant Characteristics.

	Early	Mid	Late
Age (years)	31.3 ± 4.4	31.3 ± 4.4	31.3 ± 4.4
Body Weight (kg)	79.3 ± 17.6	77.6 ± 19.6	77.3 ± 21.3
Perceived Stress Scale	11.1 ± 6.8	11.1 ± 7.4	11.3 ± 7.6
Edinburgh Postnatal Depression Scale	3.8 ± 3.4	3.7 ± 3.9	4.3 ± 4.4
**Race**			
Black	21 (24)		
White	65 (76)		
**BMI**			
Normal	19 (22)	28 (33)	29 (34)
Overweight	36 (42)	31 (36)	30 (35)
Obese	31 (36)	27 (31)	27 (31)
**Parity**			
Primiparous	32 (37)		
Multiparous	54 (63)		
**Breastfeeding**			
Exclusively	65 (75)	42 (49)	18 (21)
Some	10 (12)	16 (19)	20 (24)
None	11 (13)	28 (33)	47 (55)
**Employment**			
On maternity leave	40 (46)	1 (1)	0 (0)
Employed	22 (26)	65 (76)	60 (71)
Unemployed	24 (28)	20 (23)	24 (29)
**Education**			
High School	4 (5)	4 (5)	4 (5)
Some College	22 (25)	22 (25)	22 (25)
College Graduate	31 (36)	31 (36)	31 (36)
Graduate Degree	29 (34)	29 (34)	29 (34)
**Income**			
<$40,000	20 (23)	22 (26)	18 (21)
$40,000–$80,000	30 (35)	25 (29)	30 (35)
>$80,000	36 (42)	39 (45)	37 (44)

Data are mean ± standard deviation or *n* (%).

**Table 2:** Percent of the day spent on each of the 24-HAC behaviors.

Behavior	Early Postpartum	Mid-Postpartum	Late Postpartum
SED, %	51.0 ± 12.2	52.1± 13.0	52.3 ± 14.2
LPA, %	12.1 ± 2.8	12.9 ± 2.8	13.5 ± 3.7
MVPA, %	1.9 ± 1.1	2.0 ± 0.9	2.3 ± 1.3
Sleep, %	30.6 ± 4.5	29.6 ± 3.8	29.5 ± 3.4

Mean ± standard deviation. LPA, light physical activity. MVPA, moderate-to-vigorous physical activity. SED, sedentary behavior.

**Table 3:** Number of participants meeting Canadian Movement Behavior Guidelines.

Guideline	Early Postpartum	Mid-Postpartum	Late Postpartum
SED < 8 h/day	0 (0)	0 (0)	2 (2)
MVPA ≥ 150 min/week	49 (57)	56 (65)	66 (77)
Sleep 7–9 h/day	48 (56)	47 (55)	53 (62)

Data are presented as *n* (%).

## Data Availability

Data sharing is available upon reasonable written request to the corresponding authors and execution of a data sharing agreement.
